# Metabolic, V̇O_2_ kinetics, and muscle oxygenation responses at and above maximal lactate steady state in trained male rowers

**DOI:** 10.14814/phy2.70872

**Published:** 2026-04-24

**Authors:** Leonardo Trevisol, Fernando Klitzke Borszcz, Ricardo Dantas de Lucas, Tiago Turnes

**Affiliations:** ^1^ Physical Effort Laboratory, Sports Center Federal University of Santa Catarina Florianópolis Santa Catarina Brazil; ^2^ Department of Animal and Food Production, Agroveterinary Sciences Center Santa Catarina State University Lages Santa Catarina Brazil; ^3^ Human Performance Research Group, Center for Health and Sport Sciences Santa Catarina State University Florianópolis Santa Catarina Brazil

**Keywords:** exercise intensity domains, metabolism, NIRS, sports performance

## Abstract

This study aimed to compare physiological responses during rowing ergometer exercise at the maximal lactate steady state (MLSS) and 5% above the MLSS (MLSS+5%). Blood lactate concentration (BLC), oxygen uptake (V̇O_2_) kinetics, heart rate (HR), and muscle oxygenation (deoxygenated hemoglobin [HHb] and tissue saturation index [TSI]) of the vastus lateralis were compared. Twelve male rowers (V̇O_2_max: 53.0 ± 6.3 mL·kg^−1^·min^−1^) completed an incremental test and several 30‐min constant workload tests to determine their MLSS and MLSS+5%. At the 30th minute, exercise at MLSS+5% (195 ± 25 W) resulted in significantly higher BLC (5.02 ± 1.44 vs. 3.07 ± 1.03 mmol·L^−1^), V̇O_2_ (3.56 ± 0.35 vs. 3.33 ± 0.33 L·min^−1^), HR (177 ± 11 vs. 166 ± 10 bpm), and [HHb] (83.9 ± 16.9 vs. 72.7 ± 13.2%) compared to MLSS (185 ± 24 W) (*p* < 0.05). No V̇O_2_ slow component was observed during either the MLSS or MLSS+5% trials. In conclusion, while exercise 5% above MLSS induced a non‐sustainable BLC response, there was no apparent V̇O_2_ slow component and [HHb] remained stable in the vastus lateralis during rowing exercise.

## 1 INTRODUCTION

Exercise intensity domains can be traditionally classified as moderate, heavy, severe, and extreme (Iannetta et al., 2022; Turnes et al., 2016). During moderate‐intensity exercise, the pulmonary oxygen uptake (V̇O_2_) reaches a steady state in ~3 min, and blood lactate concentration (BLC) remains similar to resting values (Burnley & Jones, 2007). In the heavy domain, the V̇O_2_ slow component (V̇O_2SC_) represents a slow approach to a steady level of V̇O_2_, which is typically reached after 15–20 min of exercise, whereas BLC reaches a steady state (Burnley & Jones, 2007). However, during severe‐intensity exercise, BLC typically fails to reach a steady state, while V̇O_2_ is driven to maximum values during exercise of sufficient duration (De Lucas et al., 2013; Poole et al., 1988). During exercise in the extreme exercise domain, the intensity is so high that exhaustion precedes V̇O_2_max achievement (Burnley & Jones, 2007; Turnes et al., 2016). The exercise intensity domains are conventionally established by using metabolic thresholds derived from progressive or square‐wave workload tests (Bishop et al., 2025). While the transition from the moderate to heavy domains is well established and marked by the first metabolic threshold (Jamnick et al., 2020), the transition from the heavy to severe domain has been proposed as the maximal metabolic steady state (Jones et al., 2019). Currently, an intense debate exists regarding the use of the maximal lactate steady state (MLSS) for its demarcation, mainly focused on endurance sports such as cycling and running (Garcia‐Tabar & Gorostiaga, 2019; Jones et al., 2019). Although this debate is important for understanding exercise intensity domains, it is important to note that in other aerobic sports, such as rowing, the acute physiological responses around this metabolic threshold have not yet been determined. Therefore, the first step should be to investigate the acute physiological responses in the transition from the heavy to the severe domain in rowing.

Due to its distinct metabolic and biomechanical characteristics, rowing exhibits different MLSS intensity patterns compared to other aerobic modalities (Beneke, 2003a, 2003b). Indeed, a simulation‐based approach indicated that the conventional MLSS protocol tends to underestimate the point at which rates of lactate production and utilization are equal more so in rowing than in cycling (Beneke, 2003a). In this regard, studies have found MLSS at intensities lower than the second metabolic threshold (i.e., anaerobic threshold), yet higher than the first metabolic threshold (i.e., lactate thresholds), regardless of the rowers' level (Beneke, 1995; Bourdon et al., 2018; Klusiewicz, 2005; Possamai et al., 2022). Collectively, these findings demonstrate that the placement and physiological significance of MLSS within the rowing intensity spectrum remain unclear. Therefore, a prerequisite for greater clarity is to investigate the metabolic consequences of exercising around the MLSS, specifically in pulmonary V̇O_2_ on‐kinetics, BLC, and NIRS‐derived muscle oxygenation parameters.

The assessment of V̇O_2_ on‐kinetics parameters, such as the onset of the V̇O_2SC_ or V̇O_2_max attainment, is considered an important method for classifying exercise intensity domains. It is well‐established in other aerobic modalities, such as running (Nixon et al., 2021), cycling (Bräuer & Smekal, 2020; Hill et al., 2021), and swimming (Pelarigo et al., 2017), that the MLSS (defined as a BLC difference of less than 1.0 mmol·L^−1^ between the 10th and 30th minutes) does not represent the exercise intensity that determines V̇O_2_max attainment. For this goal, the severe‐intensity domain is necessary for a subject to reach V̇O_2_max. Another expected response during exercise at this intensity threshold is the emergence of the V̇O_2SC_ (Burnley & Jones, 2007). In cycling, the onset of the V̇O_2SC_ has been observed at intensities ranging from 2% below to 12% above MLSS (Hill et al., 2021), or 10 W above MLSS (Bräuer & Smekal, 2020). In contrast, in swimmers, a V̇O_2SC_ was not observed at 97.5%, 100%, or 102.5% of MLSS (Pelarigo et al., 2017). Overall, differences in muscle contraction patterns, muscle fiber recruitment profiles, and oxygen delivery (due to varying muscle mass engaged) (Poole & Jones, 2012) might explain these differences.

Specifically, in rowing, MLSS represents a lower relative intensity of the maximal work rate attained during a graded exercise test (~61%) compared to cycling and running exercise (~75%) (Borszcz et al., 2024). In addition, MLSS is further below critical power in rowing (~30% difference), irrespective of the model used, than in other whole‐body modalities (Billat et al., 2000; Borszcz et al., 2024). Thus, despite the expectation that V̇O_2_max would not be achieved around MLSS during rowing exercise, it can be hypothesized that no V̇O_2SC_ will emerge during exercise around MLSS in trained rowers, since in the severe domain the higher the muscle mass involved the larger the reduction in the V̇O_2SC_ (Billat et al., 2000). Therefore, the aim of the current study was to compare physiological responses (BLC, V̇O_2_ on‐kinetics, heart rate), as well as [HHb] and TSI from the vastus lateralis muscle, during rowing ergometer exercise at and 5% above MLSS in trained rowers.

## 2 MATERIALS AND METHODS

### 2.1 Experimental approach to the problem

Each rower completed 3–6 testing sessions within 21‐day period. All tests were separated by ≥48 h of recovery. On separate days, the rowers performed an incremental test followed by two to five square‐wave transitions used to determine MLSS and the intensity 5% above MLSS (MLSS+5%). Each participant was tested at the same time of day (±2 h) in a temperature‐controlled laboratory (22°C ± 1°C). Rowers were instructed to abstain from vigorous physical activity for 24 h before each test and to maintain their usual dietary and sleep habits during the testing period. This information was confirmed verbally before each visit.

### 2.2 Participants

An a priori sample size calculation was performed using G*Power 3.1 software (G*Power, Germany). The V̇O_2SC_ was considered the primary outcome of this study. Based on a previous study (Pelarigo et al., 2017) that reported a Cohen's *d*_*z*_ of 0.93 (mean difference divided by SD of differences) for V̇O_2SC_ between MLSS and MLSS+5%, it was determined that a sample size of 12 participants was required to achieve an alpha of 5% and a statistical power of 80%.

Twelve regional‐ to national‐level male rowers volunteered to participate in this study. The participants' characteristics were (mean ± standard deviation): age, 24 ± 10 years; height, 1.81 ± 0.05 m; body mass, 79 ± 5 kg; and training experience, 4.4 ± 3.2 years. All participants provided written informed consent; parental consent was obtained for one participant who was under the age of 18. The sample consisted of three scullers and nine sweep rowers, with five classified as lightweight and seven as heavyweights. Rowers trained 6 days per week, with a total weekly training volume of 523 ± 163 min, which included on‐water and indoor rowing training as well as resistance workouts.

This study was approved by the Institutional Ethics Committee for Research on Human Subjects (number 3.191.968) of the Federal University of Santa Catarina. The study was conducted in accordance with the Declaration of Helsinki. The participants signed informed consent before participating in the study.

### 2.3 Materials

All tests were performed on an air‐braked rowing ergometer (Concept 2E, Morrisville, VT, USA). Rowers individually set the drag factor during their first visit, and this setting was held constant for all subsequent tests (mean ± standard deviation: 125 ± 3 arbitrary units). V̇O_2_ was measured breath‐by‐breath using an automated open‐circuit gas analyzer (Quark CPET; Cosmed, Rome, Italy). The analyzer was calibrated before each test using gases of known oxygen and carbon dioxide concentrations. Capillary blood samples (25 μL) were collected from the earlobe and analyzed in duplicate for BLC using an enzyme electrode analyzer (YSI 2700, Yellow Springs, USA). Heart rate was monitored continuously throughout the tests (Polar Electro Oy, Kempele, Finland). NIRS signals were obtained from the vastus lateralis muscle using a portable, multi‐distance continuous‐wave spectroscopy device (PortaMon; Artinis Medical Systems BV, Zetten, The Netherlands) to detect relative changes in local [HHb] and TSI.

### 2.4 Exercise testing

#### 2.4.1 Incremental test

Rowers performed an incremental test until voluntary exhaustion to determine peak power output (P_PEAK_) and V̇O_2_max. The protocol began with an initial workload of 130 W for 3 min, which was then increased by 30 W every 3 min (Possamai et al., 2022). At the end of each stage, a 30‐s rest period was provided, during which capillary blood samples were collected. V̇O_2_max was defined as the highest 15‐breath rolling average, and HRmax was defined as the highest value recorded throughout the test. P_PEAK_ was defined as the power output of the last fully completed stage. If the final stage was not completed, P_PEAK_ was calculated following a linear interpolation (Possamai et al., 2022).

#### 2.4.2 Constant workload tests at MLSS and MLSS+5%

To determine MLSS, participants performed 2 to 4 constant‐work‐rate submaximal tests, each lasting 30 min, on separate days. The work rate for the first trial was set at 70% P_PEAK_ and was subsequently adjusted by 5% until MLSS was determined. MLSS precision was 10 ± 1 W (range 7 to 11 W) (Beneke, 1995; Possamai et al., 2022). Each test was preceded by a 5‐min moderate‐intensity warm‐up, followed by 5 min of passive recovery. Capillary blood samples were collected at baseline, and at the 10th and 30th minutes, during a 30‐s rest period. The MLSS was defined as the highest work rate at which BLC did not increase by more than 1 mmol·L^−1^ between the 10th and 30th minutes of the test (Beneke, 2003a). HR and V̇O_2_, as well as [HHb] and TSI from the vastus lateralis muscle, were continuously measured during the tests. For analysis, the values recorded at the 10th and 30th minutes of exercise were used.

### 2.5 V̇O_2_ on‐kinetics at MLSS and MLSS+5%

The V̇O_2_ data were first screened to exclude occasional errant breaths (e.g., from coughing, swallowing, or sighing) deemed not reflective of the underlying kinetics. Values greater than three standard deviations from the local mean were removed (Hill et al., 2021; Pelarigo et al., 2017). The V̇O_2_ data were then fitted using a nonlinear least‐squares algorithm. The mathematical models consisted of a mono‐exponential (primary component, first part of equation below) and a bi‐exponential (primary and slow components, entire equation below) function.

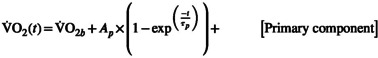

$$A_{\mathrm{sc}}\times \left(1-\exp^{\left(\frac{-t}{\tau_{\mathrm{sc}}}\right)}\right)\;\left[\text{Slow component}\right]$$

where V̇O_2_ (t) represents the absolute V̇O_2_ at time t; $\dot{V}O_{2b}$ is the V̇O_2_ during the resting baseline period; A_p_ and τ_p_ are the amplitude and time constant of the primary component; and A_sc_ and τ_sc_ are the amplitude and time constant of the slow component. Both mono‐ and bi‐exponential models were fitted to all participant data. If the bi‐exponential model failed to converge, the mono‐exponential model was used instead. In such cases, as the V̇O_2SC_ was assumed to be absent.

The V̇O_2_ on‐kinetics analysis was divided into two segments (Pelarigo et al., 2017): a “first part” (from the start of exercise to the 10th‐min break for BLC collection) and a “second part” (from the resumption of exercise to the 30th min). The delayed increase in V̇O_2_ (amplitude delayed—A_d_) was calculated as the difference between V̇O_2_ at the 30th and 10th minutes (Hill et al., 2021).

### 2.6 NIRS procedures

The NIRS raw data were recorded at 1 Hz. The probe was placed on the vastus lateralis belly, approximately halfway between the trochanter and knee joints, after the skin area had been shaved and wiped. Skinfold thickness was measured at the NIRS application site (12.3 ± 5.8 mm) using a plicometer (Lange Skinfold Caliper, Incorporated Cambridge, Maryland, USA). The device was then covered with a black, light‐absorbing cloth.

Before the incremental test, an ischemia/hyperemia calibration via arterial occlusion was performed to normalize the NIRS signals to the maximal physiological range (MPR). A pneumatic cuff was positioned on the proximal portion of the thigh and rapidly inflated to ~300 mmHg using a cuff‐inflation system (Moto Press 8.2/25 WP82251, Wyllis, Colombo, Brazil). During the procedure, participants were seated and instructed to relax and refrain from moving the leg unless requested.

### 2.7 Statistical analysis

Descriptive data are presented as mean ± standard deviation. The normality of data and model residuals was tested using the Shapiro–Wilk test. Paired‐sample Student's *t*‐tests were used to compare V̇O_2_ on‐kinetics parameters between the MLSS and MLSS+5% trials. Separate mixed‐effects models were employed to analyze BLC, HR, [HHb], and TSI responses at and above MLSS. The fixed effects were the work rate (MLSS and MLSS+5%), the time (10th and 30th minutes), and the work rate × time interaction. Participant was treated as a random effect. When appropriate, Tukey's post hoc test was used to identify pairwise differences. Two‐sided tests were used for all statistical analyses. Analyses were conducted using R (version 3.6.3; R Core Team, Vienna, Austria) via the RStudio interface (version 1.2.5; RStudio, PBC, Boston, USA). The statistical significance was set at *p* < 0.05.

## 3 RESULTS

During the incremental test, the P_PEAK_, HRmax, absolute and relative V̇O_2_max were 308 ± 39 W, 192 ± 11 bpm, 4.20 ± 0.39 L·min^−1^, and 53.0 ± 6.3 mL·kg^−1^·min^−1^, respectively. The power output at MLSS and MLSS+5% were 185 ± 24 and 195 ± 25 W, respectively. The relative power output and HR at MLSS+5% (63.4 ± 4.1%P_PEAK_; 92.5 ± 3.0%HRmax) were higher than those at MLSS (60.2 ± 3.9%P_PEAK_; 86.8 ± 3.0%HRmax; *p* < 0.001). Individual values for MLSS and MLSS+5% are presented in Table 1.

**TABLE 1:** Individual physiological responses at the 30th minute of exercise during the MLSS and MLSS+5% trials.

Rower	BLC (mmol·L^−1^)		V̇O_2_ (L·min^−1^)		HR (bpm)		[HHb] (%MPR)		TSI (%)	
	MLSS	+5%	MLSS	+5%	MLSS	+5%	MLSS	+5%	MLSS	+5%
1	4.02	6.57	—	3.80	160	176	—	102.5	58.1	48.8
2	3.60	5.88	3.87	3.74	173	177	81.5	101.5	50.5	47.2
3	1.47	3.18	2.91	3.10	174	185	56.8	73.5	61.5	60.3
4	2.64	3.42	2.89	3.32	146	153	58.8	64.4	50.2	51.5
5	2.79	3.90	3.19	3.24	158	171	58.2	62.1	52.2	55.9
6	3.48	4.86	3.44	3.82	160	169	—	—	—	—
7	2.74	5.01	3.38	—	175	188	—	—	—	—
8	1.95	4.80	3.58	3.85	158	170	87.6	108.0	53.1	53.2
9	4.50	5.94	2.99	3.26	178	187	88.5	83.6	47.6	47.3
10	4.83	8.04	3.68	3.61	165	174	—	—	—	—
11	2.65	5.16	3.24	3.43	169	185	74.5	75.0	47.1	48.2
12	2.17	3.48	3.14	3.54	181	194	75.9	84.1	54.3	53.0
Mean	3.07	4.88	3.29	3.52	166	177	72.7	81.5	52.7	51.7
SD	1.03	1.42	0.34	0.27	10	11	13.2	16.4	4.7	4.41

Abbreviations: BLC, blood lactate concentration; [HHb], deoxygenated hemoglobin concentration; HR, heart rate; MLSS, maximal lactate steady state; MPR, maximal physiological range; TSI, tissue saturation index; V̇O_2_, oxygen uptake.

**FIGURE 1:**
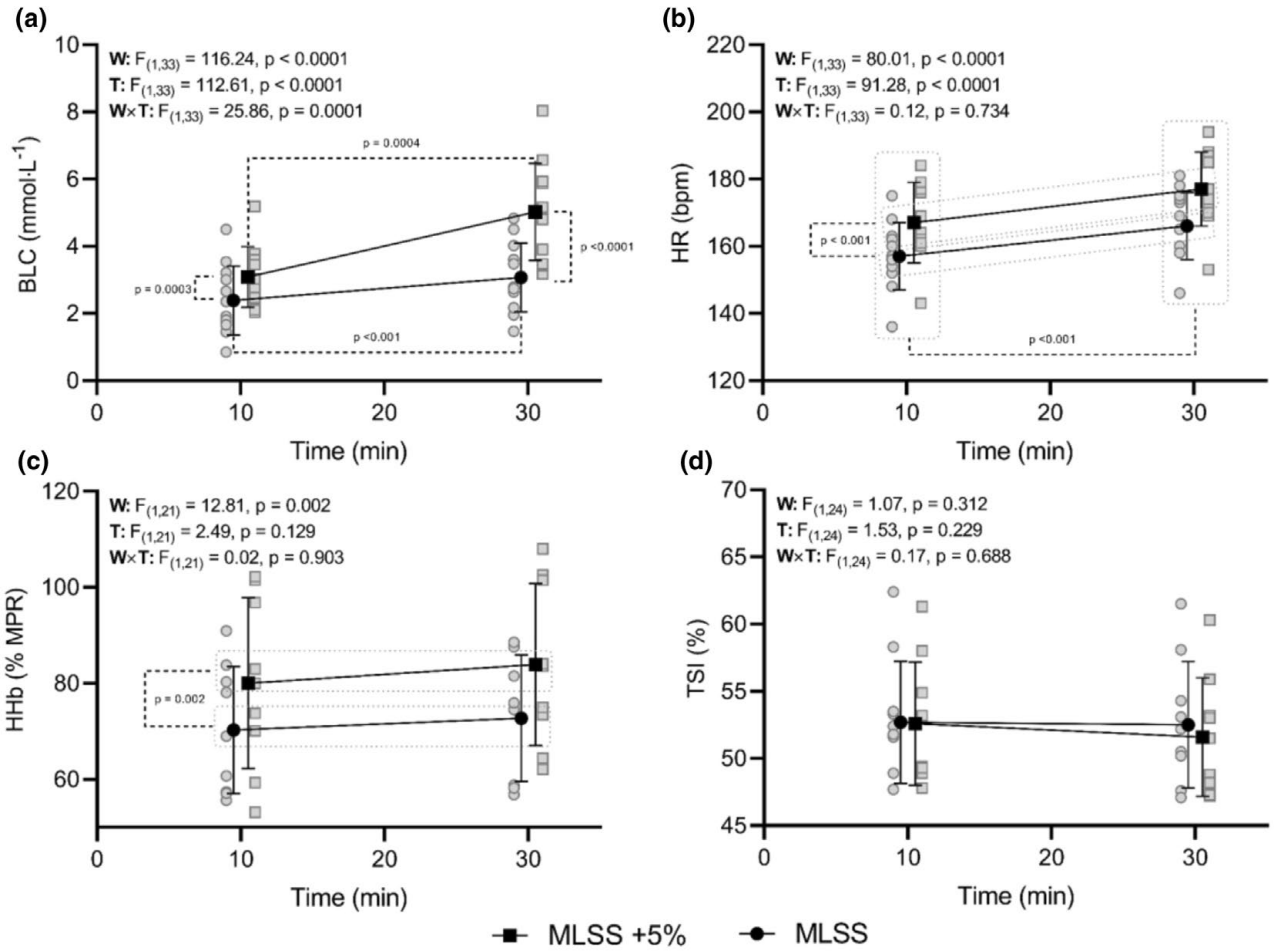
Time course of blood lactate concentration (a), heart rate (b), muscle deoxygenated hemoglobin concentration (c), and tissue saturation index (d) at and above MLSS. Values are presented as individual values (gray dots and squares), mean (black dots and squares) and standard deviation (error bars). Partial comparison *p* values for blood lactate concentration are presented comparing all time points and work rates (i.e., significant interaction). For heart rate, partial comparisons include the main effects of time and work rate. For muscle deoxygenated hemoglobin concentration, the comparison refers to the main effect of work rate.

A significant work rate × time interaction was detected for BLC (F_(1,33)_ = 25.86, *p* < 0.001) (Figure 1a). BLC significantly increased from the 10th to the 30th minute in both MLSS (*p* = 0.0004) and MLSS+5% (*p* < 0.001). However, the magnitude of this increase was different: the increase in BLC at MLSS was less than 1 mmol·L^−1^ (0.73 ± 0.25 mmol·L^−1^), while the increase in BLC at MLSS+5% was significantly greater (1.93 ± 0.62 mmol·L^−1^). Furthermore, BLC was higher at the 10th (*p* = 0.0003) and 30th (*p* < 0.0001) minutes during the MLSS+5% trial compared to the MLSS trial.

For HR (Figure 1b), there were main effects for work rate (*F*_(1,33)_ = 80.01, *p* < 0.0001) and time (*F*_(1,33)_ = 91.28, *p* < 0.0001), but no work rate × time interaction was found (*F*_(1,33)_ = 0.117, *p* = 0.734). As such, HR was higher during MLSS+5% than MLSS (work rate main effect), and it increased from the 10th to the 30th minute across both conditions (main effect of time, *p* < 0.0001) (Figure 1b).

For [HHb] (Figure 1c), there was a main effect for work rate (*F*_(1,21)_ = 12.81, *p* = 0.002), with [HHb] being higher at MLSS+5% than MLSS, but no main effect for time (*F*_(1,21)_ = 2.49, *p* = 0.129) nor a work rate × time interaction (*F*_(1,21)_ = 0.02, *p* = 0.903).

For TSI (Figure 1d), there were no main effects for time (*F*_(1,24)_ = 1.53, *p* = 0.229), work rate (*F*_(1,24)_ = 1.07, *p* = 0.312), nor work rate × time interaction (*F*_(1,24)_ = 0.17, *p* = 0.688).

**FIGURE 2:**
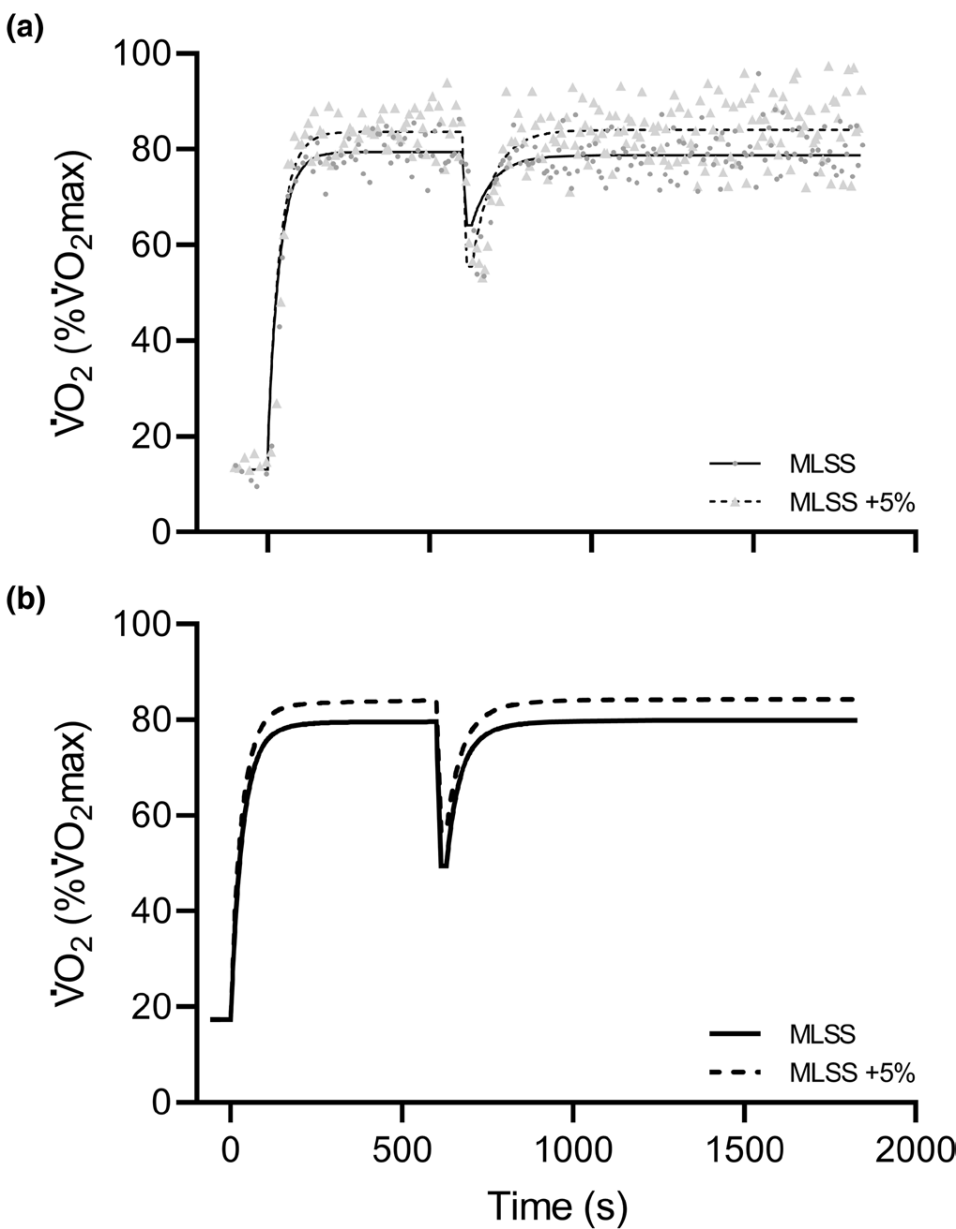
V̇O_2_ on‐kinetics during the MLSS and MLSS+5% trials for the first and second segments of exercise, showing data from a representative participant (a) and the average of all participants (b).

The V̇O_2_ on‐kinetics parameters are presented in Figure 2. In the first part (0–10 min), a bi‐exponential model converged for 3 and 4 subjects during the MLSS and MLSS+5% trials, respectively. For the remaining subjects, a mono‐exponential model was applied. In this first part, A_p_ and V̇O_2_ at the 10th minute were significantly higher at MLSS+5% (Table 2). A_SC_ values at MLSS and MLSS+5% were not different from zero (*p* = 0.170 and 0.08, respectively). In the second part (10–30 min), only the mono‐exponential model was applied, as the bi‐exponential model failed to converge for all subjects. V̇O_2b_ and V̇O_2_ at the 30th minute were significantly higher during MLSS+5%. There were no significant differences in A_d_ (i.e., V̇O_2_ difference between 10th and 30th min); furthermore, A_d_ values were not different from zero in either the MLSS (*p* = 0.476) or MLSS+5% (*p* = 0.590) condition.

**TABLE 2:** V̇O_2_ on‐kinetics parameters derived from the MLSS and MLSS+5% trials, analyzed for the first (0–10 min) and second (10–30 min) segments.

V̇O_2_ on‐kinetic parameter	Intensity		*p* Value
	MLSS	MLSS + 5%	
0–10 min			
Baseline V̇O_2_ (L·min^−1^)	0.72 ± 0.11	0.70 ± 0.11	0.591
Primary amplitude (L·min^−1^)	2.25 ± 0.32	2.72 ± 0.32*	**0.014**
Primary time constant (s)	35 ± 14	33 ± 8	0.683
V̇O_2sc_ amplitude (L·min^−1^)	0.04 ± 0.09^A^	0.12 ± 0.20^A^	0.530
V̇O_2sc_ time constant (s)	137 ± 138	458 ± 350	0.204
V̇O_2_ at 10th min (L·min^−1^)	3.32 ± 0.29	3.53 ± 0.29*	**0.008**
10–30 min			
Baseline V̇O_2_ (L·min^−1^)	2.08 ± 0.79	2.39 ± 0.56*	**0.040**
Primary amplitude (L·min^−1^)	1.06 ± 0.64	1.16 ± 0.39	0.219
Primary time constant (s)	62 ± 40	60 ± 34	0.272
V̇O_2sc_ amplitude (L·min^−1^)	0 ± 0	0 ± 0	NA
V̇O_2sc_ time constant (s)	0 ± 0	0 ± 0	NA
A_d_ (mL·min^−1^)	−15 ± 68^A^	−11 ± 65^A^	0.707
V̇O_2_ at 30th min (L·min^−1^)	3.30 ± 0.32	3.52 ± 0.27*	**0.010**
V̇O_2_ at 30th min (%V̇O_2_max)	79.9 ± 4.7	84.3 ± 5.7*	**0.009**

*Note*: Data are presented as means ± standard deviation.

*Significant difference compared to MLSS (*p* < 0.05).

^A^Not significantly different from zero (*p* > 0.05).

Abbreviations: A_d_, delayed amplitude; MLSS, maximal lactate steady state; V̇O_2SC_, V̇O_2_ slow component amplitude.

## 4 DISCUSSION

The primary novelty of this study was the investigation of physiological responses surrounding MLSS in trained rowers, a modality often overlooked compared to cycling, running, and swimming (Azevedo et al., 2021; Bräuer & Smekal, 2020; Hill et al., 2021; Iannetta et al., 2018; Nixon et al., 2021; Pelarigo et al., 2017). As expected, average physiological responses (i.e., V̇O_2_, [HHb], and HR) were higher at MLSS+5% than at MLSS. Furthermore, the MLSS+5% trial elicited a non‐sustainable BLC response, suggesting it potentially lies outside the heavy‐intensity domain. However, this finding was contrasted by the V̇O_2_ on‐kinetic responses. Apart from the expected finding that V̇O_2_max was not reached in either 30‐min trial, the more important finding was that a V̇O_2SC_ was not evident in either the MLSS or the MLSS+5% condition, which confirmed our second hypothesis. This absence of a V̇O_2SC_, even at an intensity (MLSS+5%) that was demonstrably non‐sustainable from a BLC perspective, represents a clear dissociation between metabolic and V̇O_2_ responses in rowing.

The classical model to distinguish exercise intensity domains into moderate, heavy, severe, and extreme is mainly based on V̇O_2_ on‐kinetics (Burnley & Jones, 2007). Indeed, the emergence of V̇O_2SC_ is expected at intensities above the first metabolic threshold, that is, in the heavy domain, while V̇O_2_ is driven to its maximal value into the severe domain (Jones et al., 2011). It has been extensively demonstrated that critical power represents the upper boundary from attainment or not of V̇O_2_max. Thus, given that during rowing exercise MLSS is markedly lower than critical power (Possamai et al., 2022), the finding that V̇O_2_max was not attained at either MLSS and MLSS+5% is expected and aligns with responses observed in running (Nixon et al., 2021), swimming (Pelarigo et al., 2017), and cycling (Hill et al., 2021). These data confirm that MLSS is not a metabolic marker of the transition from the heavy‐ to severe‐intensity domain in rowing exercise.

Conversely, an intriguing finding of the current study was that V̇O_2SC_ was not evident at MLSS. Even more striking was the absence of V̇O_2SC_ at MLSS+5%. This is unexpected, as the magnitude of the V̇O_2SC_ is known to increase gradually at intensities above the first metabolic threshold until reaching intensities eliciting V̇O_2_max (Poole & Jones, 2012). The current finding also differs from cycling exercise, where the V̇O_2SC_ appearance occurred at intensities slightly above MLSS (Hill et al., 2021). Additionally, a “delayed” V̇O_2SC_ was observed after 10 min at and above MLSS in cycling (Hill et al., 2021) and swimming (Pelarigo et al., 2017). While the 30‐min trial duration might contribute to such divergences, this is an unlikely explanation, as the V̇O_2SC_ is expected to emerge well before this time point (Hill et al., 2021; Pelarigo et al., 2017). Furthermore, these aspects are likely not impacted by the athletes' training level, given that elite and well‐trained rowers presented similar V̇O_2SC_ amplitudes (Ingham et al., 2007).

Within the heavy‐ and severe‐intensity domains, the V̇O_2SC_ has traditionally been associated with a progressive increase in the oxygen cost of exercise, historically attributed to reduced muscular efficiency accompanying fatigue development and the progressive recruitment of type II muscle fibers (Jones et al., 2011). However, recent evidence suggests that the decline of efficiency during heavy and severe exercise may be less pronounced than previously assumed. For instance, MacDougall et al. (2025) reported that fatigue development during high‐intensity exercise is only weakly associated with changes in efficiency, suggesting that such alterations alone may not fully explain the emergence of the V̇O_2SC_. Instead, the V̇O_2SC_ likely reflects a complex interaction of mechanisms, including muscle fiber recruitment patterns, metabolic instability, and limitations in oxygen delivery and utilization. In this context, exercise involving a larger active muscle mass has been shown to attenuate V̇O_2SC_ amplitude (Billat et al., 2000), as also observed in swimming (Demarie et al., 2001). Although rowing is primarily driven by the lower limbs, the movement requires the coordinated activation of a large proportion of the total body musculature. Therefore, it is plausible that the substantial muscle mass involved in rowing, combined with the relatively low intensity at MLSS in this modality, contributed to the absence of a detectable V̇O_2SC_ in both conditions of the present study. While the physiological mechanisms underlying the V̇O_2SC_ remain not fully elucidated, it is well established that blood lactate accumulation is not a direct cause of this phenomenon (Taboni et al., 2025). Accordingly, the present findings reinforce the dissociation between V̇O_2_ kinetics and BLC during rowing exercise, as BLC substantially increased from MLSS to MLSS+5% despite the absence of a detectable V̇O_2SC_.

Importantly, the absence of a V̇O_2SC_ in the present study should not be interpreted as evidence that this response does not occur during rowing exercise per se. Instead, several methodological and physiological factors likely explain why it was not observed under the experimental conditions employed. First, the difference between MLSS and MLSS+5% corresponded to a relatively small absolute workload change (~10 W), which may have limited the metabolic perturbation required to induce a measurable slow component. Second, the V̇O_2_ kinetics analysis was based on a single transition at each workload. It is well established that the detection of the V̇O_2SC_ is significantly improved when multiple transitions are averaged to attenuate breath‐by‐breath noise. Finally, rowing ergometer exercise is characterized by substantial movement‐related fluctuations in pulmonary gas‐exchange signals, which may further complicate the identification of low‐amplitudes slow components. This later aspect, however, might not help to explain why the V̇O_2SC_ was not detected in the present study, since a V̇O_2SC_ was detected previously in elite and club‐level rowers (Ingham et al., 2007).

Indeed, Ingham et al. (2007) observed a V̇O_2SC_ during heavy‐intensity exercise in rowers of different competitive levels. Both club‐ and Olympic‐level rowers exhibited a detectable V̇O_2SC_ during exercise at an intensity corresponding to 50% of the difference between the first lactate threshold and V̇O_2_max (i.e., ∆50%). The reported V̇O_2SC_ amplitudes were 283 ± 10 mL·min^−1^ and 541 ± 211 mL·min^−1^ for club‐level and elite rowers, respectively. Beyond the higher absolute intensities, these differences could also be attributed to greater muscle mass, as the elite rowers were significantly heavier. Notably, the ∆50% intensities in their study were positioned around the threshold of the fixed BLC of 4.0 mmol/L (e.g., club‐level: ∆50% = 269 ± 15 W vs. 4 mmol threshold = 272 ± 9 W; elite: ∆50% = 377 ± 12 W vs. 4 mmol threshold = 391 ± 11 W). Since ∆50% is located near the upper boundary of the heavy‐intensity domain, it is a common benchmark for eliciting a V̇O_2SC_. Conversely, and as expected, Ingham et al. (2007) did not observe a V̇O_2SC_ during moderate‐intensity exercise. It is also noteworthy that while those authors employed multiple transitions (two for heavy and four for moderate intensity) to refine their kinetic analysis, each transition lasted only 6 min, which may constrain the full emergence of the V̇O_2SC_. In contrast, the 30‐min duration of our protocol allowed for a more prolonged observation of metabolic stability. Therefore, it can be argued that neither the training status nor the number of transitions was the primary factor for the absence of a V̇O_2SC_ in the current study. Instead, our findings suggest that in rowing, the MLSS likely represents an intensity toward the lower boundary of the heavy‐intensity domain, which may not be sufficient to trigger a detectable V̇O_2SC_.

With regard to muscle oxygenation, it has been reported that the muscle oxygen extraction limit is reached in the heavy domain in runners (Rodrigo‐Carranza et al., 2021), cyclists, and triathletes (Vasquez Bonilla et al., 2023). In the current study, the [HHb] response at MLSS+5% was not different from the peak values observed during the incremental test. This finding aligns with previous data suggesting similarities between MLSS and [HHb] breaking‐point intensities in rowers (Possamai et al., 2024). Although these oxygenation responses might indicate that MLSS marks the attainment of the oxygen extraction limit for the *vastus lateralis* during rowing exercise, caution is required as this may simply represent a steady‐state [HHb] response. In contrast, we found no significant difference in TSI between the MLSS and MLSS+5% conditions. This lack of difference may be explained by known methodological limitations; during high‐intensity exercise, TSI results can be confounded by tissue ischemia and movement artifacts from more frequent muscle contractions (Crum et al., 2017). Furthermore, literature shows that at intensities representing 84% of the second lactate threshold, only small differences in TSI between the 10th and 30th minute of exercise have been reported (Klusiewicz et al., 2021). Taken together, these results suggest that [HHb] is a more sensitive tool than TSI for detecting small differences in exercise intensity around MLSS, and it remains a valuable marker for training prescription.

Although V̇O_2_ reportedly stabilized after 20 min of exercise at and above MLSS in cycling (Jones et al., 2011), in the current study, HR increased continuously in both trials, and the delayed V̇O_2_ (i.e., V̇O_2SC_) was not observed. This delayed V̇O_2_ stabilization indicates that not all exercise intensities that exceed the heavy‐severe boundary will lead to V̇O_2_max (Jones et al., 2011). This is consistent with our findings, as V̇O_2_max was not reached in either trial. However, the difference in V̇O_2_ behavior (i.e., delayed stabilization in cycling versus absence of V̇O_2SC_ in rowing) despite potentially similar HR responses may be explained by the greater muscle mass involved in rowing. This imposes higher demands on the cardiorespiratory and central nervous systems. Additionally, considering the unique physiological and biomechanical characteristics of rowing, along with the fact that second metabolic threshold clearly overestimates MLSS work rate (Klusiewicz et al., 2021), the present results are crucial for a deeper understanding of the significance of MLSS in rowing exercise. Further research should explore these findings during rowing exercise performed to exhaustion, as well as a possible association with maximal metabolic steady‐state, to better understand the exercise intensity domains during rowing ergometer exercise.

The observed BLC at MLSS in rowers may be primarily attributed to the specific motor pattern and the large active muscle mass typical of this modality. Unlike cycling or running, rowing involves approximately 85% of the total body muscle mass through the coordinated action of the legs, trunk, and arms (Beneke, 2003a). This extensive muscle involvement suggests that, for a given oxygen uptake, the power output and metabolic demand per unit of muscle mass are lower than in modalities engaging smaller muscle volumes (Beneke, 2003a; Beneke et al., 2001; Beneke & von Duvillard, 1996). Supporting this, glycogen depletion at similar relative intensities has been shown to be lower in the legs than in the arms (Ahlborg & Jensen‐Urstad, 1991), and glycogen utilization in the legs is significantly reduced when arm exercise is added to cycle ergometer (Richter et al., 1988). Together, these findings help explain the higher MLSS levels observed during arm cranking and speed skating compared to cycling or running, which are notably higher than those observed in rowing (Ahlborg & Jensen‐Urstad, 1991; Beneke, 2003a; Beneke et al., 2001; Beneke & von Duvillard, 1996). Furthermore, the specific force dynamics of the rowing stroke, characterized by high‐intensity intermittent contractions, influence the rates of glycolysis and pyruvate oxidation (Beneke et al., 2001). These biomechanical factors, combined with the “lactate shuttle” mechanism, facilitate a dynamic equilibrium between lactate formation and clearance at lower systemic concentrations, typically around 3.0 mmol/L^−1^ in rowers (Beneke & von Duvillard, 1996; Possamai et al., 2022, 2024). These findings are of practical importance as they suggest that traditional fixed‐concentration thresholds (e.g., 4.0 mmol·L^−1^) likely overestimate the MLSS in rowers. Consequently, the distinctive interaction between substantial muscle mass and stroke‐specific force profiles may explain why MLSS levels in rowers do not strictly follow the classic patterns observed in other whole‐body exercises.

Despite the originality of investigating exercise around MLSS in rowing, the current study has limitations that should be addressed. First, the V̇O_2_ on‐kinetics analysis was performed with only a single transition at each workload. Second, due to the movement pattern, V̇O_2_ data measured during rowing ergometer is known to exhibit a lower signal‐to‐noise ratio, which may have impacted the data analysis. Moreover, the 30‐s recovery period for BLC measurement during the MLSS and MLSS+5% trials might have influenced V̇O_2_ behavior due to partial recovery. This aspect, along with the exercises not being performed to exhaustion, must be acknowledged. However, both data selection and treatment were carried out using an individualized and manual approach to enhance overall accuracy, thereby increasing confidence in the results obtained.

Furthermore, methodological considerations must be discussed. Although the coefficient of variation for the MLSS determination (12.7%) was slightly higher than the 10% recommendation (Garcia‐Tabar & Gorostiaga, 2018), which carries a minor risk of underestimating the power output, this discrepancy was considered small. The present study used a fixed percentage above MLSS instead of the fixed work rate, a design choice implemented to ensure that athletes exercised just above their individual MLSS. Finally, a primary criticism of MLSS concerns the reliability of BLC measurements (Jones et al., 2019). In the current study, all BLC samples were analyzed in duplicate using a YSI 2700 Biochemistry Analyzer (YSI Incorporated, Yellow Springs, OH, USA), which features a manufacturer‐reported coefficient of variation below 2%. Additionally, Hauser et al. (2013) reported a coefficient of variation for BLC at MLSS of 17%, whereas MLSS intensity presented a value corresponding to 3%. It is important to emphasize that MLSS assessment relies on the change in BLC during exercise rather than absolute values per se. Consequently, MLSS determination exhibits low day‐to‐day variability (3%), suggesting that BLC kinetics are a sensitive and appropriate metric for confident MLSS assessment (Garcia‐Tabar & Gorostiaga, 2019). Therefore, the rigorous MLSS determination protocol employed herein (Table S1) enhances the reliability of the metabolic responses observed at and slightly above the MLSS threshold.

In conclusion, a slight increment in intensity above MLSS induced substantial increases in BLC, HR, V̇O_2_, and [HHb] compared to exercise at MLSS in trained rowers. These findings indicate that during rowing ergometer exercise, the V̇O_2SC_ is dissociated from BLC, as V̇O_2SC_ was not evident during exercise above MLSS, despite a concomitant non‐sustainable BLC response.

## AUTHOR CONTRIBUTIONS

**Leonardo Trevisol:** Conceptualization; investigation; methodology. **Fernando Klitzke Borszcz:** Conceptualization; investigation; methodology. **Ricardo Dantas de Lucas:** Conceptualization. **Tiago Turnes:** Conceptualization; investigation; methodology; supervision.

## FUNDING INFORMATION

This work was supported by *Coordenação de Aperfeiçoamento de Pessoal de Nível Superior* – Brazil (CAPES) – Finance code 001.

## CONFLICT OF INTEREST STATEMENT

None declared.

## ETHICS STATEMENT

This study was approved by the Institutional Ethics Committee for Research on Human Subjects (number 3.191.968) of the Federal University of Santa Catarina. The study was conducted in accordance with the Declaration of Helsinki. All the participants signed informed consent before participating in the study.

## Supporting information


Table S1.

## Data Availability

The data supporting the findings of this study are available from the corresponding author upon reasonable request.
