# Supplementary material for: Metabolic, V̇O2 kinetics, and muscle oxygenation responses at and above maximal lactate steady state in trained male rowers
Source: Physiol Rep. 2026 Apr 24;14(8):e70872. doi: 10.14814/phy2.70872 (PMC13109653; doi:10.14814/phy2.70872)
Supplement: Supplementary file 1 — Table S1. [file PHY2-14-e70872-s001.docx]

**Physiological Reports**

**Metabolic, V̇O_2_ Kinetics, and Muscle Oxygenation Responses at and Above Maximal Lactate Steady State in Trained Male Rowers**

Leonardo Trevisol^1^, Fernando Klitzke Borszcz^1,2,3^, Ricardo Dantas de Lucas^1^, Tiago Turnes^1^

**Affiliations:**

^1^Physical Effort Laboratory, Sports Center, Federal University of Santa Catarina, Florianópolis, SC, Brazil.

^2^Department of Animal and Food Production, Agroveterinary Sciences Center, Santa Catarina State University, Lages, SC, Brazil.

^3^Human Performance Research Group, Center for Health and Sport Sciences, Santa Catarina State University, Florianópolis, SC, Brazil.

**Corresponding author:**

Tiago Turnes. Physical Effort Laboratory, Sports Center, Federal University of Santa Catarina, Florianópolis, Brazil. E-mail: tiago.turnes@ufsc.br

**Supplemental Material File**

**Supplementary table 1.** Individual identification of MLSS.

|  | **MLSS trial 1** | | | | **MLSS trial 2** | | | | **MLSS trial 3** | | | | **MLSS trial 4** | | | |
| --- | --- | --- | --- | --- | --- | --- | --- | --- | --- | --- | --- | --- | --- | --- | --- | --- |
|  | **PO** | **BLC (mmol/L)** | | | **PO** | **BLC (mmol/L)** | | | **PO** | **BLC (mmol/L)** | | | **PO** | **BLC (mmol/L)** | | |
|  | **(W)** | **10^th^** | **30^th^** | **∆** | **(W)** | **10^th^** | **30^th^** | **∆** | **(W)** | **10^th^** | **30^th^** | **∆** | **(W)** | **10^th^** | **30^th^** | **∆** |
| 1 | 200 | 1.92 | 2.78 | 0.86 | 210 | 3.06 | 2.97 | -0.09 | **220** | **3.21** | **4.02** | **0.81** | 231 | 3.78 | 6.57 | 2.79 |
| 2 | 186 | 3.12 | 4.02 | 0.90 | **195** | **3.00** | **3.60** | **0.60** | 205 | 3.6 | 5.88 | 2.28 |  |  |  |  |
| 3 | 159 | 3.09 | 5.16 | 2.07 | 151 | 2.35 | 3.78 | 1.43 | 143 | 2.04 | 3.18 | 1.14 | **136** | **0.85** | **1.47** | **0.62** |
| 4 | 190 | 3.09 | 6.15 | 3.06 | 180 | 2.25 | 3.42 | 1.17 | **171** | **2.35** | **2.64** | **0.29** |  |  |  |  |
| 5 | 190 | 3.30 | 4.59 | 1.29 | 180 | 2.77 | 3.90 | 1.13 | **171** | **1.91** | **2.79** | **0.88** |  |  |  |  |
| 6 | 190 | 2.77 | 4.86 | 2.09 | **180** | **2.66** | **3.48** | **0.82** |  |  |  |  |  |  |  |  |
| 7 | 213 | 4.02 | 8.01 | 3.99 | 202 | 3.03 | 5.01 | 1.98 | **192** | **1.76** | **2.74** | **0.98** |  |  |  |  |
| 8 | 204 | 1.65 | 2.21 | 0.56 | **214** | **1.45** | **1.95** | **0.50** | 225 | 2.47 | 4.80 | 2.33 |  |  |  |  |
| 9 | **174** | **3.54** | **4.5** | **0.96** | 183 | 3.63 | 5.94 | 2.31 |  |  |  |  |  |  |  |  |
| 10 | 204 | 2.37 | 3.09 | 0.72 | **214** | **4.50** | **4.83** | **0.33** | 225 | 5.19 | 8.04 | 2.85 |  |  |  |  |
| 11 | 186 | 3.45 | 5.16 | 1.71 | **177** | **1.80** | **2.65** | **0.85** |  |  |  |  |  |  |  |  |
| 12 | 186 | 2.11 | 3.48 | 1.37 | **177** | **1.66** | **2.17** | **0.51** |  |  |  |  |  |  |  |  |

Notes: Data in **bold** represent the identification of MLSS for this subject. PO = power output. BLC = Blood lactate concentration. 10^th^ = BLC at minute 10. 30^th^ = BLC at minute 30. ∆ = BLC difference from minute 30th to 10th.
